# The Evaluation of Thyroid Diseases in Patients with Pemphigus Vulgaris

**DOI:** 10.1100/2012/146897

**Published:** 2012-10-15

**Authors:** Mukaddes Kavala, Esra Kural, Emek Kocaturk, Ilkin Zindanci, Zafer Turkoglu, Burce Can

**Affiliations:** Department of Dermatology, Istanbul Medeniyet University Goztepe Training and Research Hospital, Istanbul, Turkey

## Abstract

*Background*. Thyroid disorders may affect all of the organ systems of the body and they are also highly associated with a wide variety of skin disorders. The aim of this study was to investigate the prevalence of thyroid function abnormalities and thyroid autoimmunity in patients with pemphigus vulgaris (PV) and to determine the association between thyroid disorders and clinical involvement and systemic corticosteroid treatment in patients with PV. *Methods*. The study consisted of eighty patients with PV and eighty healthy individuals. Thyroid functions (fT3, fT4, and TSH) and thyroid autoimmunity (anti-thyroid peroxidase (anti-TPO), and anti-thyroglobulin (anti-Tg) antibodies) were investigated in both groups. Primary thyroid disease (PTD) was diagnosed with one or more of the following diagnostic criteria: (i) positive antithyroid antibodies, (ii) primary thyroid function abnormalities. *Results*. Significant changes in the serum thyroid profile were found in 16% (13/80) of the PV group and 5% (4/80) of the control group. Positive titers of antithyroid antibodies (anti-TPO and anti-Tg) were observed in 7 patients (9%) with PV and one in the control group (1,2%). Hashimoto thyroiditis was diagnosed in 9% of PV patients and it was found to be more prevalent in the mucosal form of PV. PTD was found in 13 of (%16) PV patients which was significantly high compared to controls. PTD was not found to be associated with systemic corticosteroid use. Free T3 levels were significantly lower in PV group compared to the control group and free T4 levels were significantly higher in PV group compared to the controls. *Conclusions*. PV may exist together with autoimmune thyroid diseases especially Hashimoto thyroiditis and primer thyroid diseases. Laboratory work-up for thyroid function tests and thyroid autoantibodies should be performed to determine underlying thyroid diseases in patients with PV.

## 1. Introduction

Pemphigus is an autoimmune bullous disorder which is characterized by the formation of IgG-type autoantibodies against desmosomal adhesion molecules [1]. There is a common association between thyroid diseases and skin disorders such as melasma, vitiligo, alopecia, Sjögren's syndrome, idiopathic hirsutism, connective tissue disorders and bullous diseases [2]. Dermatitis herpetiformis and thyroid diseases are reported to be strongly associated; 34% of the patients were found to have hypothyroidism, Graves' disease, toxic multinodular goiter, and follicular carcinoma [3]. Pemphigus vulgaris (PV) is accompanied by some autoimmune disorders and a recent study has demonstrated increased susceptibility to the development of autoimmune diseases in the family members of patients with PV [4]. Nevertheless there are still a few studies evaluating thyroid autoimmunity in patients with PV [5, 6]. We aimed to investigate the presence of primary thyroid diseases by analyzing serum thyroid profiles in patients with PV. 

## 2. Patients and Methods

A total of 80 consecutive PV patients and 80 age, and sex-matched healthy control subjects were enrolled in the study. Exclusion criteria were presence and history of thyroid disease both in the patient and in patient's family, systemic diseases such as diabetes and malignancy, and usage of drugs that interfere with thyroid functions for the last 6 months. Forty-seven of PV patients were under systemic corticosteroid therapy while 33 were new patients without therapy. Serum thyroid profiles of patients and controls were evaluated with analyzing thyroid function tests and thyroid autoantibodies. Serum-free triiodothyronine (fT_3_), free thyroxine (fT_4_), thyroid stimulating hormone (TSH), anti-thyroid peroxidase (anti-TPO) and anti-thyroglobulin (anti-Tg) antibodies were measured by electrochemiluminescence assay (Elecsis 170 modular analyzer, Roche Diagnostics). The diagnosis of primary thyroid disease was confirmed by the presence of anti-thyroid antibodies and/or abnormalities in thyroid function tests. 

NCSS 2007 (Number Cruncher Statistical System) and PASS 2008 statistical software programme, student's *t*, chi-square, and Fisher's Exact tests were used for the statistical analysis of the data. *P* < 0.05 was taken as statistically significant. 

## 3. Results 

The female/male ratio was 54/26 in both study and control groups (67.5% female, 32.5% male), the average age was 52.5 in PV group and 51.5 in control group. The duration of the disease was 52.4 with a range between 1 month and 168 months. Forty-seven (58,8%) of PV patients were receiving 5–125 mg/day prednisolon, while 33 (41,3%) were new patients without therapy. Thirty-nine (48%) of the patients had mucosal, 15 (19%) had cutaneous, and 26 (33%) had both mucosal and cutaneous involvement. Thirteen (16%) of PV patients and 4 (5%) of control group had alterations in serum thyroid profiles (thyroid function tests and anti-thyroid antibodies); this finding was statistically significant (*P* < 0.05). Ten (12.5%) of PV patients and 4 (5%) of the controls were found to have abnormalities in thyroid function tests and the difference between groups was statistically significant (*P* < 0.05). In PV group, levels of fT3 were significantly lower and fT4 levels were significantly higher when compared to control group (*P* < 0.05). There was no significant difference with respect to TSH levels between patients and controls (*P* > 0.05). Seven (9%) of PV patients and 1 (1.2%) of controls were found to have anti-thyroid antibodies (anti-TPO and/or anti-Tg) and the difference between groups were statistically significant (*P* < 0.05). Anti-TPO antibodies were found in 6 (%8) and anti-Tg antibodies were found in 2 (2,5%) of PV patients. In the control group 1 (1.2%) patient had both anti-TPO and anti-Tg antibodies. The frequency of anti-TPO antibodies were significantly higher in PV group than the control group (*P* < 0.05), while the frequency of anti-Tg antibodies showed no significant difference between groups (*P* > 0.05) (Table 1). 

Primary thyroid disease (PTD) was found in 13 (16%) of the patients: subclinical Hashimoto thyroiditis in 7 (9%), subclinic hyperthyroidism in 2 (2,5%), subclinic hypothyroidism in 3 (3,7%), and euthyroid syndrome in 1 (1%). Six of the patients with Hashimoto thyroiditis had anti-TPO, one had anti-Tg, and one had both anti-TPO and anti-Tg while 4 had alterations in thyroid function tests (Table 2). Four (5%) individuals in the control group were found to have PTD. One of them (1%) had subclinical Hashimoto thyroiditis, 1 (1%) had subclinical hyperthyroidism, and 2 (2.5%) had subclinical hypothyroidism. The one with Hashimoto thyroiditis had alterations in thyroid functions (Table 3). There was a significant difference between patients and control group with respect to presence of PTD (*P* < 0.05) and PV group had a higher frequency of PTD. In addition, the frequency of Hashimoto thyroiditis was higher in PV group than the control group (*P* < 0.05).

Of the 13 PV patients with PTD 6 (46%) were receiving 5–40 mg/day prednisolone while 7 (54%) were without treatment. We found no association between steroid usage and presence of PTD in PV patients (*P* > 0.05). Of the PV patients with PTD, 5 (38.5%) had mucosal, 3 (23%) had cutaneous, and 5 (38,5%) had both cutaneous and mucosal involvement; mucosal involvement was found more prevalent in PV patients with PTD but there was no significant difference when compared with PV patients (*P* > 0.05). Hashimoto thyroiditis was found in 4 (%30.8) patients with mucosal involvement, in 1 (7.7%) patient with cutaneous involvement, and in 2 (15.4%) patients with both cutaneous and mucosal involvement. PV patients with Hashimoto thyroiditis had a higher frequency of mucosal involvement but this finding was statistically insignificant (*P* > 0.05). Of the patients with Hashimoto thyroiditis 2 were receiving systemic corticosteroids and 5 were without treatment; we found no significant association between Hashimoto thyroiditis and systemic steroid usage (*P* > 0.05).

## 4. Discussion

Autoimmune disorders may accompany each other and co-existence of PV with autoimmune disorders such as myasthenia gravis, systemic lupus erythematosus, rheumatoid arthritis, and Graves' disease has been reported [7]. In addition, autoimmune thyroid disorders have been reported in association with PV [3, 8, 9]. In our study we found alterations in thyroid function tests and thyroid autoantibodies in 16% of PV patients and 5% of the controls. The alterations in serum thyroid profiles were significantly higher in PV patients. Our findings were similar to the findings of Pitoia et al. who screened 15 PV patients with respect to thyroid diseases [5]. Pitoia et al. found abnormalities in serum thyroid profiles in 47% of PV patients and 7% of the controls. The authors found anti-TPO autoantibodies in 40% of PV patients and 7% of the controls; only one of the patients with anti-thyroid antibodies had Hashimoto thyroiditis. Ansar et al. found anti-TPO antibodies in 23% of 22 PV patients and in 6% of the controls, they found no thyroid diseases in none of the patients with positive anti-thyroid antibodies [6].

Similar to the recent studies, we found higher prevalance of anti-thyroid antibodies (anti-TPO and anti-Tg) in PV patients (9% of PV patients and 1% of the controls). But we found subclinical Hashimoto thyroiditis in all of the PV patients with positive anti-thyroid antibodies. Former studies reported only presence of anti-thyroid antibodies but they found no abnormalities in thyroid function tests [5, 6]. We found thyroid function abnormalities in a higher number of patients in PV group than the control group and we observed that average fT3 levels were significantly lower and average fT4 levels were significantly higher in PV patients than the control group. 

Our study differed from the other two studies because we found PTD in all of the patients who were found to have serum thyroid profile alteration and PV patients were found to have higher prevalence of PTD when compared to placebo [5, 6]. In addition, PV and Hashimoto thyroiditis were found to have a significant association and Hashimoto thyroiditis was more common in the mucosal form of PV even though this finding was not statistically significant. Pitoia et al. found subclinical hypothyroidism in 7% of PV patients, while Ansar et al. did not find any thyroid disease which is associated with abnormalities of thyroid functions [5, 6]. We found subclinical hypothyroidism in 4%, subclinical hyperthyroidism in 3%, and euthyroid syndrome in 1% of PV patients and all of these patients had alterations in thyroid function tests. We did not find any association between PTD and clinical phenotype of PV and gender of the patients. 

Both of the former studies reported that study patients were under low- or moderate-dose systemic corticosteroid treatment and suggested that corticosteroids could suppress thyroid autoimmunity and might be the reason of the lower amounts of anti-thyroid antibodies than expected [5, 6]. Systemic corticosteroids are known to suppress autoimmune reactions by attenuating T cell proliferations [10]. They are used in the treatment of Hashimoto thyroiditis where they act to lower the titers of thyroid autoantibodies and normalize thyroid functions [11]. It has been reported that systemic corticosteroids could affect thyroid autoimmunity in a dose-dependent manner and high-dose corticosteroids could suppress while lower doses could accelerate thyroid diseases [8]. Niepomniszcze et al. reported that in patients with endogeneous Cushing's syndrome, the normalization of hypercortisolism triggers the occurrence of autoimmune thyroid diseases in susceptible individuals [12]. Takasu et al. also reported that in patients with thyroid autoimmunity, normalization of hypercortisolism exacerbates autoimmune phenomenon and leads to thyroid diseases [13]. We found similar frequencies of PTD in patients with or without systemic corticosteroid therapy and we observed that corticosteroids did not effect thyroid autoimmunity and lower doses of corticosteroids did not increase occurrence of thyroid diseases [8, 12, 13]. 

Firooz et al. reported that the frequency of thyroid diseases had a threefold increase in the first-degree relatives of PV patients and suggested that genetic susceptibility is responsible for the occurrence of more than one autoimmune disease in an individual [4]. Bartalena et al. claimed that the high frequency of HLA DR_3_ and HLA D_4_ both in PV and Graves' disease emphasizes the role of genetic predisposition in these two diseases [8].

As a conclusion, even though the exact causes remain unidentified, the results of our study showed that PV could accompany thyroid autoimmunity and primary thyroid diseases especially Hashimoto thyroiditis. We recommend laboratory testing for thyroid autoantibodies and thyroid function tests in patients with PV even if they do not have a clinical indication of thyroid disease. 

## Figures and Tables

**Table 1:** Levels of anti-thyroid antibodies and thyroid function tests in patients with PV and control subjects.

	PV patients		Control subjects		P^+^
	Mean	SD	Mean	SD
fT3 (pg/mL)	3.23	0.68	3.45	0.61	0.037
fT4 (ng/mL)	1.42	0.95	1.20	0.24	0.043
TSH (uIU/mL)	2.30	2.26	2.33	1.48	0.94
Anti-TG (IU/mL)	65.23	77.15	71.90	125.66	0.69
Anti-TPO (IU/ mL)	28.62	70.12	11.35	20.14	0.036

Normal values: TSH: 0.27–4.2 uIU/mL; ft3: 2.5–4.3 pg/mL; ft4: 0.93–1.7 ng/mL; Anti TG: 0–115 IU/mL; Anti-TPO: 0–34 IU/ML.

^+^Student's *t* test.

**Table 2:** Thyroid functions and anti-thyroid antibody levels in PV patients with thyroid disease.

Patient	TSH	TSH	fT3	fT3	fT4	fT4	Anti-TPO	Anti-TPO	Anti-TG	Anti-TG
4	N	2.75	N	2.98	N	1.05	H	45.6	N	17,5
6	H	14.25	N	0.98	N	9.50	N	7.28	N	10.00
9	N	1.38	N	2.86	N	1.01	N	5.00	H	562.00
13	N	3.60	N	3.12	N	1.02	H	243.00	N	52.00
25	L	0.19	H	2.70	Y	1.75	N	8.82	N	10.30
50	N	1.49	N	3.12	D	0.89	H	42.52	N	15.35
51	L	0.21	N	2.84	N	1.35	N	7.23	N	17.95
52	H	6.55	N	2.90	D	0.89	N	5.89	N	10.00
56	H	13.46	L	1.80	D	0.80	H	587.00	H	400.00
59	N	1.84	L	1.29	N	1.17	N	5.60	N	10.00
60	N	0.66	L	2.40	N	1.28	H	34.26	N	10.66
78	L	0.07	L	1.95	N	1.53	N	5.00	N	13.24
79	H	3.70	N	3.53	N	1.27	H	147.00	N	13.28

H: High, N: Normal, L: Low.

**Table 3:** Thyroid functions and anti-thyroid antibody levels in control subjects with thyroid disease.

Subject	TSH	TSH	fT3	fT3	fT4	fT4	Anti TPO	Anti TPO	Anti TG	Anti TG
27	H	7.57	N	3.32	N	1.05	H	101.7	H	1141.00
75	L	0.15	L	2.15	N	1.29	N	7.21	N	10.00
76	L	0.15	H	5.50	H	2.70	N	2.12	N	38.23
77	H	7.43	N	2.76	N	1.13	N	33	N	46.40

H: High, N: Normal, L: Low.
